# From Sprinters to Marathoners: Rethinking Therapeutic Speed and Disease Modification in Atopic Dermatitis

**DOI:** 10.1111/all.70395

**Published:** 2026-05-18

**Authors:** Thomas Bieber, Felix Lauffer

**Affiliations:** ^1^ Department of Dermatology and Allergy Ludwig‐Maximilians University of Munich Munich Germany; ^2^ Christine Kühne‐Center for Allergy Research and Education, Medicine Campus Davos Davos Switzerland; ^3^ Bieber Dermatology Consulting Feldafing Germany

**Keywords:** atopic dermatitis, biomarkers, disease modification, drug development, remission

The therapeutic landscape of atopic dermatitis (AD) has expanded at a pace unmatched in its prior decades [1]. Yet, as new agents enter clinical practice, it is increasingly clear that efficacy measured at one predefined time point alone does not fully capture their clinical identity. The *tempo* of response—how quickly a therapy exerts meaningful clinical benefit on signs and symptoms—was for a long time a defining characteristic. This temporal dimension not only shapes patient expectations and adherence but also reflects deeper mechanistic differences with implications for long‐term disease modification. The classical time point at week 16 (as defined in the context of the clinical development program (CDP) of dupilumab more than a decade ago) for defining the primary endpoints for clinical response has long been accepted as the standard to which drug developers, regulatory agencies, and investors were sticking to compare the efficacy of established drugs and those in CDP. However, this leads “to compare apple and oranges” as the speed and type of response are tightly linked to the respective mode of action. A useful metaphor is borrowed from athletics: JAK inhibitors (JAKi) as sprinters, current biologics as mid‐distance runners, and emerging pathway‐resetting agents such as amlitelimab and rezpegaldesleukin as marathon runners. Each class runs the same race—reducing inflammation, itch, and improving quality of life—but they do so with distinct pacing strategies and their intrinsic strengths, benefit/risk ratios, and potentials to control the disease on the long term are different (Table 1).

**TABLE 1:** Summary of the characteristics of the speed‐classes.

Speed Class	Mode of action	Examples	Onset	Durability	Most common adverse events
Sprinters	Blocking signaling of multiple cytokines (including IFN‐γ, IL‐4, IL‐5, IL‐10, IL‐13, IL‐31) by targeting Janus Kinase 1 and 2 IL‐31 receptor inhibition	Baricitinib Upadacitinib Abrocitinib Nemolizumab (especially itch)	Very fast (within days on pruritus)	Quick relapse after cessation	Upper respiratory tract infections, acne, headache, nausea. Caution in patients over 65, smokers, those with cancer, and those with a history of thrombosis (applies only to JAK inhibitors)
Mid‐Distance Runners	Blocking IL‐4 and/or IL‐13 signaling	Dupilumab Tralokinumab Lebrikizumab	Moderately fast (within 2–4 weeks)	Gradual decline of effectiveness after discontinuation	Conjunctivitis
Marathon Runners	Blocking OX40 signaling Stimulating expansion of regulatory T cells via high affinity IL‐2 receptor	Amlitelimab Rezpegaldesleukin	Slow onset of action but continued improvement beyond week 24	Signals of a long‐lasting response even after discontinuation	Pyrexia, Headache, herpes virus infections, conjunctivitis (applies only to OX40 inhibitors) Injection site reactions, fever, eosinophilia, headache (applies only to Rezpegaldesleukin)

The Sprinters: JAKi and the Era of Rapid Suppression. JAKi such as upadacitinib, abrocitinib, and baricitinib have redefined what “rapid onset” means in AD [2]. Their mechanism—intracellular blockade of JAK–STAT signaling—interrupts multiple cytokine pathways simultaneously, including IL‐4, IL‐13, IL‐31, TSLP, and interferons. This broad, proximal inhibition produces: (i) Pruritus reduction within days, sometimes within 24 to 48 h; (ii) EASI improvements visible by week 1–2 and consequently and (iii) high rates of early patient‐reported symptom relief. Like elite sprinters, JAKi excel in fast mode of action with immediate symptom control, particularly for patients with severe itch, sleep disruption, or acute flares. However, sprinters expend energy quickly. This is evident from the fact that relapses occur quickly after discontinuation, and disease‐specific biomarkers (such as TARC/CCL17) do not show a reduction but, in some cases, even an increase during therapy [3]. This intriguing phenomenon could be explained at least in part by a compartmentalized and persistent epidermal immune dysregulation despite clinical improvement. Long‐term safety considerations require careful patient selection and ongoing vigilance. Interestingly, at least in terms of reducing itch nemolizumab can be assigned to this group. However, its effect on skin inflammation seems to be substantially related to the concomitant use of topical anti‐inflammatory drugs such as glucocorticosteroids and/or calcineurin inhibitors.

The Mid‐Distance Runners: the biologics Dupilumab, Tralokinumab, Lebrikizumab as Steady, Reliable Performers [4]. Biologics targeting the IL‐4/IL‐13 axis represent the middle distance: fast enough to be clinically meaningful early yet paced for sustained control. Their pharmacology is more selective than JAKi. They either inhibit the type 2‐specific effector cytokine IL‐13 or, in the case of dupilumab, also inhibit the Th2 cell differentiation cytokine IL‐4. Their clinical profile reflects this: (i) onset typically within 2 to 4 weeks, with progressive improvement thereafter; (ii) durable disease control under therapy, often with plateauing efficacy after about 6 months; and (iii) strong long‐term safety and adherence profiles. These agents run with rhythm and consistency but the clinical response is highly variable and unpredictable. They may not match the explosive start of JAKi, but they deliver predictable, sustained, and safe disease control and achieve similar goals, just a little later.

The Marathon Runners: Amlitelimab [5] and Rezpegaldesleukin [6] and the Prospect of Disease Modification [7]. A new category is emerging therapies whose initial pace is slower but whose long‐term trajectory suggests deeper immunologic recalibration. By targeting the OX40–OX40L axis, amlitelimab modulates T‐cell co‐stimulation without depleting cells. When considering the putative immunological cascade involved in the generation of the inflammatory reaction in AD [1, 2], it is assumed that the more upstream the pharmacological target is located (such as OX40L expressed on cutaneous dendritic cells), the longer it will take to obtain a clinical efficacy. On the other hand, such pharmacological intervention bears the potential to deeply impact on the T cell differentiation, to expand the Treg population and to potentially erase the tissue memory for inflammation. Indeed, clinical data show: (i) gradual onset compared with JAKi or IL‐4/IL‐13 blockers; (ii) continued improvement beyond week 16, extending into week 24 and beyond; (iii) signals of durable remission after treatment cessation in early studies. A confirmation of these preliminary results will be needed in further phase 3 and post‐marketing studies. Nevertheless, safety signals must be closely monitored, as the mechanism of action is not specific to type 2 immunity and distinct safety signals have led to stop the CDP of rocatinlimab. By expanding regulatory T cells, rezpegaldesleukin aims to restore immune tolerance rather than merely suppress inflammation. Early trials demonstrate: (i) slower early clinical response; (ii) progressive improvement with sustained dosing; and (iii) potential for long‐lasting immune rebalancing. These agents resemble marathon runners: they build momentum gradually, but their strength lies in endurance and the possibility of altering the disease course itself. If confirmed in phase 3 programs, they may represent the first true disease‐modifying therapies in AD—an aspiration long held but never realized.

The “speed classes” metaphor is more than a rhetorical device. It reflects fundamental immunologic principles: Patients differ in what they need: some require immediate relief such as a patient presenting with a sudden, severe flare of AD, with intense pruritus, sleep disruption, and widespread erythema. The priority is rapid symptom control to restore function and quality of life.

In this situation, a fast‐acting therapy—a “sprinter” —is particularly valuable. A drug with rapid onset can quickly reduce itch and inflammation, preventing escalation to systemic corticosteroids or emergency care. In contrast, others prioritize long‐term stability such as patients with long‐standing, moderate‐to‐severe AD with a stable but persistent disease burden. They are less concerned about rapid improvement and more focused on long‐term safety and durable disease modification. Here, a slower onset therapy—a “marathoner”—may be ideal if it offers deeper immunologic modulation and sustained control over months to years.

As the field matures, dermatology may shift from a paradigm of symptom suppression to one of immune restoration. The arrival of marathon‐class agents forces us to rethink treatment goals: should early speed remain the dominant metric of success? Or should we value therapies that may reduce disease activity years into the future? How do we counsel patients when the fastest drug is not necessarily the one that best aligns with their long‐term goals?

The future of AD management may involve combined or sequencing strategies—sprinters for rapid control, mid‐distance runners for stabilization, and marathoners for long‐term immune remodeling. However, this kind of therapeutic scenarios will require the attention of the third‐party payers such as the Health Technology Assessment Agencies in Europe.

The therapeutic race in AD is no longer a single event but a multi‐stage competition. JAK inhibitors, IL‐4/IL‐13 biologics, and emerging immune‐resetting agents each bring unique strengths. It is expected that, besides data issued from controlled trials in the respective CDP, evidence generated from available and future real‐world data will help to recognize these differences and allow clinicians to tailor therapy not only to disease severity but also to patient priorities, immunologic phenotype, and long‐term aspirations. If drugs such amlitelimab and rezpegaldesleukin ultimately demonstrate disease‐modifying potential, the marathon may become the most important race of all since this is would finally fulfill the expectations of almost all patients suffering from AD and their relatives.

## Funding

This work was supported by Christine Kühne–Center for Allergy Research and Education.

## Conflicts of Interest

T. Bieber is/was speaker and/or consultant for AbbVie, Aclaris, Almirall, AnaptysBio, Apogee, Aristea, Attovia, Belenos, Biocryst, Biogeneration, BioVerSys, Bluefine, Böhringer‐Ingelheim, Bristol‐Myers Squibb, BYOME Labs, Cantargia, CellDex, Choate, Dermavant, DirigentBio, Domain Therapeutics, EMD Serono, EngImmune, Ennovate, Evommune, Forbion, Formycon, Galderma, Gilead, GSK, ImagGeneBio, Janssen, Kyowa/Kirin, Kymera, LEO, LG Chem, Lilly, Mabylon, MSD, Micreos, Nektar, Nextech, Novartis, Numab, Ornavi, Overtone, Pfizer, Pierre Fabre, Protagonist Tx, Samsung Bioepis, Sanofi/Regeneron, Scienta Lab, Serono, SwitchKine, TIRmed, Triton Therapeutics, Triveni, UCB, Union Therapeutics, UPStream Bio, WinWard, YUHAN, Zurabio. He is founder and chairman of the board of the non‐profit biotech “Davos Biosciences AG” within the international Kühne‐Foundation. He is also the founder and senior medical advisor of the consulting firm “Bieber Dermatology Consulting”.

F. L is/was speaker and/or consultant for Abbvie, Almirall, Amgen, Biogen, Boehringer Inglheim, Bristol‐Myers‐Squibb, Janssen, Johnson & Johnson LEO Pharma, Lilly, Incyte, Novartis, Roche, Sanofi, Regeneron, UCB, Union Therapeutics, Hexal, Biogen.

## Data Availability

Data sharing not applicable to this article as no datasets were generated or analyzed during the current study.
